# Ego-Resiliency and Perceived Social Support in Late Childhood: A Latent Growth Modeling Approach

**DOI:** 10.3390/ijerph18062978

**Published:** 2021-03-14

**Authors:** Qishan Chen, Wenyang Gao, Bin-Bin Chen, Yurou Kong, Liuying Lu, Shuting Yang

**Affiliations:** 1 School of Psychology, Center for Studies of Psychological Application, and Guangdong Key Laboratory of Mental Health and Cognitive Science, South China Normal University, Guangzhou 510631, China; gunslly@163.com; 2 School of Management, University of Science and Technology of China, Hefei 230026, China; gaowy@mail.ustc.edu.cn; 3 Department of Psychology, Fudan University, Shanghai 200433, China; chenbinbin@fudan.edu.cn; 4 School of Management, Xiamen University, Xiamen 361005, China; kongyurou@m.scnu.edu.cn; 5 Beijing Key Laboratory of Applied Experimental Psychology, National Demonstration Center for Experimental Psychology Education, Faculty of Psychology, Beijing Normal University, Beijing 100875, China; ystpsy@163.com

**Keywords:** late childhood, ego-resiliency, perceived social support, latent growth modelling

## Abstract

This study explored the change trajectory of schoolchildren’s ego-resiliency and perceived social support and investigated the effect of perceived social support on ego-resiliency across four time points. A sample of 437 children aged 8–13 years (*M* = 10.99, *SD* = 0.70, 51.5% boys) completed assessments at four time points. The results indicated that ego-resiliency showed an increasing linear trend and perceived social support showed a declining linear trend. Perceived social support had a positive effect on ego-resiliency over time. In addition, the initial status of perceived social support negatively predicted the growth trend of ego-resiliency, and the initial status of ego-resiliency negatively predicted the declining trend of perceived social support. The implications for theory and practice are discussed.

## 1. Introduction

Ego-resiliency is the dynamic capacity to modify one’s own level of ego-control according to situational contexts [1]. Ego-control refers to the “threshold or operating characteristic of an individual with regard to the expression or containment of impulses, feelings, and desires” [2]. Both ego-resiliency and ego-control are key factors in understanding motivation, emotion, and behavior [1,3]. Individuals high in ego-resiliency easily change their level of ego-control, adapt to changing situations, and perform flexible problem-solving strategies, while individuals low in ego-resiliency have difficulty modifying their level of ego-control, tend to show anxiety, and show little adaptive flexibility [2,4,5,6].

A large body of research has explored the development of ego-resiliency, and most of the existing research has focused on particular age stages. Among them, Block and Block [4] examined the development of ego-resiliency from toddlerhood to young adulthood. Chuang, Lamb, and Hwang [7] studied ego-resiliency’s development from early childhood to adolescence. Some studies have focused on the development of ego-resiliency during the toddlerhood and preschool periods [8,9], some have focused on early childhood [10,11], and others have focused on the period from late adolescence to young adulthood [12,13]. In the present study, we focused on late childhood, which is the transition period in psychological development from childhood to early adolescence [14]. Children in the transition period are vulnerable to negative events. Ego-resiliency has been shown to play a crucial role during the transition period, which provides the resources necessary to self-regulate academic behaviors [15]. Ego-resiliency has also been associated with social competence [9,16] and low levels of internalizing and externalizing behaviors [7,17]. Therefore, exploration of the development of ego-resiliency during this period will be beneficial to prevention and intervention work. This present study examines the change trajectory of ego-resiliency and perceived social support during late childhood and explores the potential relationships between perceived social support and ego-resiliency across four time points during late childhood.

### 1.1. Change Trajectory of Ego-Resiliency

Many researchers have revealed the different developmental trends of ego-resiliency. For example, Taylor et al. [9] found ego-resiliency’s growth trend in children from the ages of three and a half to seven. Chuang and colleagues [7] found a declining trend of ego-resiliency between the ages of seven and eight. Alessandri et al. [12] found that ego-resiliency’s development was stable until the end of high school, and later, an increasing trend from the ages of 19 to 25 was observed. These findings suggest that ego-resiliency shows particular change tendencies at different age stages. Developmental transitions may be the sources of changes in ego-resiliency [2] because individuals have to adapt to changes in their environment and social expectations [15]. The period from late childhood to early adolescence (approximately ages 9 to 11) is an important transition in cognitive and emotional development [18,19]. Ego-resiliency is assumed to be affected by other personality characteristics and learning, such as emotionality and coping skills [8]. As a personality characteristic, effortful control has an important impact on ego-resiliency. Effortful control refers to “the efficiency of executive attention, including the ability to inhibit a dominant response and/or to activate a subdominant response, to plan, and to detect errors” [20], and is closely related to ego-resiliency. Previous studies have found that effortful control has a positive effect on ego-resiliency [17,21,22]. In the early years of life, effortful control develops quickly [17,20], which may foster the development of ego-resiliency. However, with the gradual maturation of cognitive and emotional abilities from late childhood to early adolescence, children may have increased ego-resiliency [15]. Previous research has shown that children can easily gain the resources to cope with stressors from family during middle childhood [23]. Some researchers found that the use of problem-solving skills showed an increasing trend during the period from childhood to early adolescence [18,24], which may foster the development of ego-resiliency. Therefore, we propose that during the time period of this study, the trajectory of the children’s ego-resiliency will show an increasing trend (hypothesis 1).

### 1.2. Change Trajectory of Perceived Social Support

Two major aspects of social support are received and perceived social support. Received social support refers to the actual support behaviors provided by networks. Perceived social support is defined as the subjective perceptions or beliefs that one can receive social support from others when necessary. Individuals with high perceived social support are prone to seek external resources [25]. Compared with received social support, perceived social support has been regarded as having a more consistent relationship with social adaptation [26,27].

Perceived social support is a potentially important factor during childhood, a period of social vulnerability and mental reliance in multiple settings (e.g., family, school, and peer groups). Research on children’s perceived social support typically focuses on emotional support, informative support or advice, instrumental or material support, and social companionship [28]. Different supportive relationships provide different kinds of social support [29]. Researchers have demonstrated that children have a well-differentiated sense of their social support network [30]. They know that they have distinctive social needs and that different types of support can be met by different people [31]. Children perceive their mothers as the best available multipurpose social support provider compared to friends and teachers. Friends are perceived as the best source of companionship support and emotional support. Teachers are perceived as good providers of informational support or advice. Fathers are also rated as excellent sources of informational support.

Individuals seek specific types of social support in their relationships with others [32]. For example, schoolchildren experience a shift in life and learning from family to school. The size of the children’s social support network becomes larger than before, and the available supportive behaviors from the network also change. As children move into middle childhood, parent–child attachment remains critical for children’s development. In particular, the availability of parents becomes more important than proximity [33]. Availability refers to open communication between the parent and child and the parents’ responses to the child’s needs [34]. In addition to social support from family, children spend the most time in school during middle childhood. Teachers and peers are important components of children’s social relationships. Teachers play a powerful and influential role in children’s lives [35]. Teacher–child relationships provide a resource for children’s development [36]. A supportive teacher–child relationship will help children receive a good learning experience [37]. Additionally, peer relationships are salient during the adolescent years [30]. Positive peer relationships play a great role in children’s development [38]. Children have been shown to perceive the same amount of social support from parents and friends in late childhood [39].

Considering changes in perceived social support sources, such as attachment relationships with their parents, teacher–child relationships, and peer relationships, we propose that during the time period of this study, the trajectory of children’s perceived social support will show an increasing trend (hypothesis 2).

### 1.3. Reciprocal Relationships between Perceived Social Support and Ego-Resiliency

Ego-resiliency as a dynamic capacity can be acquired and shaped over time in response to environmental demands [40]. According to the ecological systems theory [41], family and school are microsystems that have a direct effect on children’s development. Because the scope of children’s lives is mainly family and school, ego-resiliency may be shaped by protective factors from family and school. On the one hand, parental support behaviors can meet children’s psychological needs for autonomy and competence [42]. Existing evidence has shown that perceived supportive parental behaviors are positively related to ego-resiliency [43,44,45]. On the other hand, research has indicated that positive peer and teacher–student relationships play vital roles in fostering resilience [35]. With increased interactions with teachers and peers, children may develop increasingly resilient behaviors by modeling and internalizing adaptive behaviors, such as problem-solving skills [35]. The existing literature suggests that received social support is positively related to resiliency. We thus infer that perceived social support may be positively associated with ego-resiliency. As previously mentioned, perceived social support has a more important effect on children’s social adaptation. When facing difficulties, children with a high level of perceived social support believe that they can receive social support from others. These beliefs may help them gain more confidence to solve problems. Even without the help of others, they may succeed in solving problems. Therefore, perceived social support may be positively related to ego-resiliency. However, the level of perceived social support relies on actual support behaviors from others. That is, when children possess enough actual social support, perceived social support may promote the development of ego-resiliency.

Ego-resiliency, as a personality trait [2], may influence perceived social support. Personality traits affect perceived social support by influencing how perceived supportive behaviors are received, eliciting different responses from others and fostering individuals’ positive behaviors in accordance with their personalities [46]. Compared with ego-brittle children, ego-resilient children, typified by a high-level of confidence and optimism, easily elicit a supportive response from others [47]. Ego-resilient children have positive relations with others [48]. In the face of challenging or threatening circumstances, they are likely to seek out social resources [49]. Researchers have found that ego-resiliency predicts perceived social support [50]. Therefore, it further suggests that ego-resiliency may be positively related to perceived social support.

Exploring bidirectional longitudinal relationships between ego-resiliency and perceived social support is particularly worthwhile because, to our knowledge, there has been no research done on such relationships during late childhood. Hill et al. [32] found that the reciprocal relationships between the initial levels of the Big Five personality traits effect different aspects and changes in perceived social support in adulthood. It is possible that other personality characteristics and perceived social support interact with each other. Therefore, we predict that perceived social support may be positively associated with ego-resiliency at every time point, that the initial status of perceived social support at Time 1 may be positively associated with the rate of increasing ego-resiliency, and that the initial status of ego-resiliency at Time 1 may be positively associated with the rate of increasing perceived social support (hypothesis 3).

In the present research, we investigated relationships between ego-resiliency and perceived social support using a four-wave longitudinal design. First, we examined the change trajectory of ego-resiliency and perceived social support across four time points. Then, we examined how perceived social support would be related to ego-resiliency.

## 2. Materials and Methods

### 2.1. Participants

A group of primary school students in Guangdong Province, China, participated in the present study. Cluster random sampling was used. Data were first collected in December 2015 (Time 1 (T1)), and the data from other waves (Time 2 (T2), Time 3 (T3), and Time 4 (T4)) were collected every 6 months. A total of 681 students finished self-report questionnaires at T1. Of those, we received 617 usable questionnaires. Among them, 47.8% were in year 3, and 52.2% were boys (*M*_age_ = 8.87 years, *SD* = 0.71). A total of 591 of the T1 students finished the survey at T2 (96%), 541 of the T2 students finished the survey at T3 (92%), and 437 of the T3 students finished the survey at T4 (81%). Attrition was mainly because (1) some participants had transferred to another school and (2) some students were absent from school during the assessment. We compared students who completed measures across all waves with students who dropped out of at least one wave. There was no significant difference in ego-resiliency (*t* = −0.836, *p* > 0.05), perceived social support (*t* = 1.013, *p* > 0.05), or gender (*χ*^2^ = 0.295, *p* > 0.05). The missing completely at random (MCAR) test was used to clarify the trend of the missing data [51]. The result was not significant (*χ*^2^/*df* = 1, *p* > 0.05), suggesting that the data were missing at random. The final sample of 437 participants had 51.5% boys, and 33.2% were in year 4 (*M*_age_ = 10.99 years, *SD* = 0.70).

### 2.2. Procedure

Before conducting the study, we obtained approval from the Research Ethics Committee of the respective university. All participants and their parents provided consent forms before completing each assessment across the four time points. Trained teachers conducted every survey in the participants’ classrooms. Participants were instructed to complete all the measures and then provided their demographic information (i.e., age, gender, and grade) at every time point. At the beginning of each assessment, the trained teachers read and explained the standardized instructions to guide students to complete the assessments. The participants were told that the data were collected and analyzed anonymously. The students could take as long as they needed to finish the survey.

### 2.3. Measures

Ego-resiliency. Ego-resiliency was measured by the ego-resiliency scale [47], which consists of fourteen items. Responses were given on a five-point scale (1 = strongly disagree to 5 = strongly agree). An example item is “I quickly get over and recover from being startled”. The coefficient alpha at every time point was 0.71, 0.76, 0.78, and 0.80.

Perceived social support. Perceived social support was measured by the adapted version [52] of the perceived social support scale [53]. It consisted of twelve items, and an example item is “My family is willing to help me make decisions”. This scale was scored on a 5-point scale (1 = strongly disagree to 5 = strongly agree). It included three dimensions: family support, support from friends, and support from other significant people. Here, we aggregated all item scores to form a composite score. The values of the coefficient alpha of the whole scale at every time point were 0.80, 0.85, 0.85, and 0.89.

### 2.4. Analytic Strategies

We used latent growth modelling (LGM) to model the change trajectories of ego-resiliency and perceived social support [54]. First, we selected an optimal model from the considered models (including linear and quadratic growth models). For the linear growth model, the factor loading of the latent intercept factors was set to 1 to reflect the initial status. The factor loading of the latent slope factors was fixed at 0, 1, 2, and 3 for four repeated measures, representing linear growth. For the quadratic growth model, in addition to intercept factors and linear slope factors, quadratic slope factors were fixed to the square values of linear slope factors [55]. Then, based on the best-fitted model, we considered the effect of time-varying covariates (i.e., perceived social support) on the change trajectory of ego-resiliency. Finally, we specified a parallel process latent growth model to test the relationship between the change trend of perceived social support and the change trend of ego-resiliency [55,56]. The parallel process latent growth model has been widely applied in many areas of psychology [57,58,59,60].

Descriptive statistics and correlations for all study variables were calculated using SPSS Version 21.0. All latent growth models were conducted using Mplus Version 7.4. Model fit was evaluated by five fit indices: chi-square to degrees of freedom ratio (*χ*^2^/*df*), the Tucker–Lewis Index (TLI), the comparative fit index (CFI), the root mean square error of approximation (RMSEA), and the standardized root mean square residual (SRMR). A *χ*^2^/*df <* 3, a CFI and TLI of > 0.90, and an RMSEA and SRMR of *<* 0.08 are considered indications of good model fit [61].

## 3. Results

### 3.1. Descriptive Statistics

Descriptive statistics and bivariate correlations among all observed variables are presented in Table 1. The results indicated that ego-resiliency was positively correlated with perceived social support from T1 to T4. Given the significant correlations between gender and perceived social support, we used gender as a control variable in the latent growth model.

### 3.2. Latent Growth Models for Ego-Resiliency and Perceived Social Support

Table 2 summarizes the model fit indices of latent growth models for ego-resiliency and perceived social support. Table 3 provides parameter estimates of latent growth models for ego-resiliency and perceived social support. For ego-resiliency, both the linear growth model and the quadratic growth model fit the data very well (for the linear growth model, *χ*^2^(*df*) = 13.454(5), *χ*^2^/*df* = 2.691, CFI = 0.985, TLI = 0.982, RMSEA = 0.062, and SRMR = 0.044; for the quadratic growth model, *χ*^2^(*df*) = 3.009(1), *χ*^2^/*df* = 3.009, CFI = 0.996, TLI = 0.978, RMSEA = 0.068, and SRMR = 0.016). Because the linear growth model is nested under the quadratic growth model [54], we compared ego-resiliency’s linear growth model and quadratic growth model by using the chi-square test. The result was significant (Δ*χ*^2^ = 10.45, Δ*df* = 4, and *p* = 0.03). Although the linear growth model and quadratic growth model for ego-resiliency were significantly different, the slope factors of the quadratic growth model were not statistically significant, so we used the linear growth model of ego-resiliency. The rate of change (slope factor) was positive and significant (μ_S_ = 0.35, *p* < 0.05), suggesting that ego-resiliency increased linearly across the time period of this study. Therefore, hypothesis 1 was confirmed. The slope factor variances were statistically significant (σ^2^_S_ = 6.31, *p* < 0.001), indicating that there were individual differences in the rate of change. The intercept factor variances were statistically significant (σ^2^_I_ = 41.84, *p* < 0.001), revealing that there were individual differences in initial status at Time 1. Finally, the factor covariance between the intercept and the slope for ego-resiliency was negative and statistically significant (σ_IS_ = −0.40, *p* < 0.001), which indicates that students who had a higher level of ego-resiliency at Time 1 increased in ego-resiliency at much slower rates over time than those with a lower level at Time 1.

For perceived social support, the fit indices of both the linear and quadratic growth models were acceptable (for the linear growth model, *χ*^2^(*df*) = 21.018(7), *χ*^2^/*df* = 3, CFI = 0.975, TLI = 0.964, RMSEA = 0.068, and SRMR = 0.085; for the quadratic growth model, *χ*^2^(*df*) = 3.899(2), *χ*^2^/*df* = 1.950, CFI = 0.997, TLI = 0.983, RMSEA = 0.047, and SRMR = 0.011). In the quadratic growth model of perceived social support, a nonpositive definite problem appeared in the latent variable covariance matrix. The quadratic slope of the quadratic growth model was statistically nonsignificant, so we retained the linear growth model of perceived social support. The rate of change (slope factor) was negative and significant (μ_S_ = −0.81, *p* < 0.001), suggesting that perceived social support decreased linearly across the time period of this study. Hypothesis 2 was not supported. The slope factor variances were statistically significant (σ^2^_S_ = 6.58, *p* < 0.001), indicating that there were individual differences in the rate of change. The intercept factor variances were statistically significant (σ^2^_I_ = 40.70, *p* < 0.001), revealing that there were individual differences in the initial statuses at Time 1. In addition, gender was negatively related to the intercept (initial status) of perceived social support (γ = −0.16, *p* < 0.01), showing that girls perceived more support than boys at Time 1. Finally, the factor covariance between the intercept and the slope for perceived social support was negative and statistically significant (σ_IS_ = −0.33, *p* < 0.001), which indicates that students who had a higher level of perceived social support at Time 1 experienced smaller decreases in perceived social support over time than those with a lower level at Time 1.

### 3.3. Linear Growth Model for Ego-Resiliency with a Time-Varying Covariate

Based on a linear growth model for ego-resiliency, we tested the roles of perceived social support on ego-resiliency at every time point. The model showed acceptable fit (*χ*^2^(*df*) = 53.535(17), *χ*^2^/*df* = 3.149, CFI = 0.958, TLI = 0.946, RMSEA = 0.070, and SRMR = 0.092). Perceived social support was positively related to ego-resiliency at every repeated measure (T1: γ = 0.38, *p* < 0.001; T2: γ = 0.40, *p* < 0.001; T3: γ = 0.37, *p* < 0.001; T4: γ = 0.37, *p* < 0.001), which suggests that perceived social support had a stable influence on ego-resiliency.

### 3.4. Parallel Process Latent Growth Model for Ego-Resiliency and Perceived Social Support

**Figure 1:**
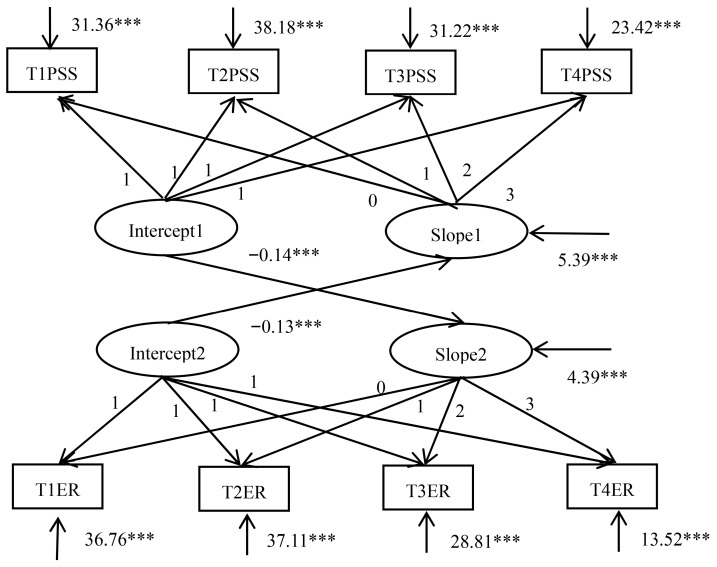
Parallel process latent growth model for ego-resiliency and perceived social support. Notes. PSS = perceived social support, ER = ego-resiliency, *** *p* < 0.001.

On the basis of a linear growth model for ego-resiliency and perceived social support, we specified a parallel process latent growth model. Figure 1 provides the results of the parameter estimates. The model had acceptable fit (*χ*^2^(*df*) = 127.740(24), *χ*^2^/*df* = 5.323, CFI = 0.927, TLI = 0.915, RMSEA = 0.099, and SRMR = 0.079). The intercept factor of perceived social support was negatively related to the slope factor of ego-resiliency (γ = −0.14, *p* < 0.001), which shows that students who perceived a higher level of social support at Time 1 increased in ego-resiliency at much slower rates over time. The intercept factor of ego-resiliency was negatively related to the slope factor of perceived social support (γ = −0.13, *p* < 0.001), which indicates that students who had a higher level of ego-resiliency at Time 1 decreased in perceived social support at much faster rates over time. Based on the results above, hypothesis 3 was partly supported.

## 4. Discussion

The present study explored the change trajectory of ego-resiliency and perceived social support across four time points, as well as the positive effect of perceived social support on ego-resiliency. We also examined the reciprocity between ego-resiliency and perceived social support. School children’s ego-resiliency increased during the time period of the current study, while perceived social support decreased. However, perceived social support had a positive influence on ego-resiliency at every time point. Furthermore, the initial status of ego-resiliency negatively predicted the declining trend of perceived social support, and the initial status of perceived social support negatively predicted the growth trend of ego-resiliency.

As we hypothesized, ego-resiliency showed a growth trend over time. As an adaptation ability, ego-resiliency showed an increasing trend in late childhood. This suggests that the developmental trajectories in different developmental stages may have unique patterns. For example, Alessandri et al. [12] found that the developmental window of ego-resiliency was age 19. We found that ego-resiliency significantly changed over time, at least during late childhood. For preadolescents, cognitive and emotional competences were gradually developing to cope with the challenges in their lives. Their frequent interactions with teachers and peers in school may promote their ability to solve a variety of problems. Therefore, their ego-resiliency may increase with age.

However, perceived social support showed a declining trend, which was inconsistent with our hypothesis. One recent study also demonstrated that children exhibited lower support seeking tendencies in late childhood [62]. At the end of primary school, students experience the transition from childhood to adolescence, characterized by independence and autonomy. In addition, during middle childhood, children often face an imbalance between unrealistic expectations of success and real ability levels, and receive more failure feedback [14]. This may lead them to becoming more independent and having a lower subjective perception of social network support. Consequently, they may feel less social support from others. In addition, the difference in perceived social support across genders is consistent with previous findings [63]. Girls are more likely to seek social support than boys [64]. Compared to boys, girls are more likely to show self-disclosure and emotional expressiveness [65], which may enhance social interactions where girls are more willing to seek help and perceive more social support than boys.

Our findings indicated that perceived social support predicted ego-resiliency. Thus, hypothesis 3 was partly supported. During late childhood, children spend most of their time with family and at school. Although they perceived lower levels of social support from family and school, social support still played an important role in their development of ego-resiliency. Children can readily gain external help from parents, teachers, and peers when facing troubles. Protection from immediate family members can foster ego-resiliency [2]. In addition, support from teachers and peers may be associated with ego-resiliency [35]. Our findings confirmed the protective role of perceived social support on ego-resiliency. In addition, we found that the initial status of ego-resiliency increased the decline rate of perceived social support. Highly resilient individuals often have a high level of self-confidence and good psychological adjustment [47]. Ego-resilient children possess abundant interpersonal skills [2], which may play a crucial role in gaining social support. Ego-resilient children may perceive declining social support due to their high level of self-confidence in gaining social support. Interestingly, the initial status of perceived social support inhibited the growth rate of ego-resiliency. This means that excessive social support is not always beneficial to the development of ego-resiliency, especially during late middle childhood. Interpersonal relationships play a dual role in adolescents; they not only provide social support but also bring stressors, such as parents’ attention to academic performance and acceptance by peers [66]. Social support that meets recipients’ actual needs may be more practical and acceptable. In addition to received and perceived social support, there are different kinds of classifications. As noted above, Cohen and Wills [28] divided social support into four categories: emotional support, social companionship, instrumental support, and informative support. Different kinds of social support may play different roles in ego-resiliency. Camara et al. [66] found that emotional support is the most important support for adolescents when facing stress events. The effects of different types of social support on ego-resiliency should be explored in the future.

This study has several limitations that need to be addressed in future research. First, although we used a longitudinal design to explore the causal relations between ego-resiliency and perceived social support, change in the short-term is easily affected by unexpected factors. Future research should attempt to use a more long-term longitudinal design to gain clear causality. For example, studies exploring the whole primary school stage may provide more information about the developmental trajectory. Second, self-report measures were used in the current study. This may increase common method variance. Therefore, future research should also include other sources of ratings, such as parents, teachers, and peers. Finally, we focused on only one protective factor for ego-resiliency. There are multiple factors that may promote ego-resiliency, including family factors such as parenting styles [22] and children’s individual factors such as the Big Five personality traits [67].

## 5. Conclusions

By using a latent growth modelling approach, we found a linear increasing change in ego-resiliency and a linear declining change in perceived social support. Perceived social support had a positive effect on ego-resiliency at every time point. The initial status of perceived social support negatively predicted the increasing rate of ego-resiliency. The initial status of ego-resiliency negatively predicted the declining rate of perceived social support. This research suggests that children’s ego-resiliency may be influenced by perceived social support during late childhood when it is often prone to decline.

## Figures and Tables

**Table 1:** Descriptive Statistics and Correlations.

Variables	M	SD	1	2	3	4	5	6	7	8	9
1. Gender	0.51	-	1								
2. Ego-resiliency(T1)	48.01	8.48	−0.04	1							
3. Ego-resiliency(T2)	48.78	8.76	−0.02	0.53 **	1						
4. Ego-resiliency(T3)	48.49	8.26	−0.03	0.33 **	0.49 **	1					
5. Ego-resiliency(T4)	49.17	8.37	−0.04	0.33 **	0.49 **	0.68 **	1				
6. Perceived Social Support(T1)	45.85	8.24	−0.12 *	0.44 **	0.36 **	0.18 **	0.21 **	1			
7. Perceived Social Support(T2)	44.09	9.09	−0.10 *	0.26 **	0.50 **	0.36**	0.34**	0.51 **	1		
8. Perceived Social Support(T3)	44.04	8.70	−0.11 *	0.20 **	0.30 **	0.44 **	0.38 **	0.37 **	0.58 **	1	
9. Perceived Social Support(T4)	43.42	9.49	−0.08	0.21 **	0.31 **	0.42 **	0.53 **	0.29 **	0.51 **	0.63 **	1

*n* = 437; T = Time; 1 = boys, 0 = girls, the mean represented the proportion of boys. * *p* < 0.05, and ** *p* < 0.01.

**Table 2:** Model Comparisons for Ego-Resiliency and Perceived Social Support.

Models	χ^2^/*df*	CFI	TLI	RMSEA	SRMR
Ego-resiliency					
Linear growth model	2.691	0.985	0.982	0.062	0.044
Quadratic growth model	3.009	0.996	0.978	0.068	0.016
Perceived social support					
Linear growth model	3.000	0.975	0.964	0.068	0.085
Quadratic growth model	1.950	0.997	0.983	0.047	0.011

**Table 3:** Parameter Estimates of Latent Growth Models for Ego-Resiliency and Perceived Social Support.

Models	Means of Growth Factors			Variances of Growth Factors		
	Intercept Factor	Linear Factor	Quadratic Factor	Intercept Factor	Linear Factor	Quadratic Factor
Ego-resiliency						
Linear growth model	48.09 ***	0.35 *		41.84 ***	6.31 ***	
Quadratic growth model	48.02 ***	0.35	0.007	70.56 ***	45.32 ***	2.17 *
Perceived social support						
Linear growth model	46.55 ***	−0.81 ***		40.70 ***	6.58 ***	
Quadratic growth model	46.84 ***	−1.49 *	0.23	58.10 ***	38.83 **	1.06

*n* = 437; gender was considered a control variable for the latent growth model of perceived social support. * *p* < 0.05, ** *p* < 0.01, and *** *p* < 0.001.

## Data Availability

Data sharing is not applicable to this article.
